# Sulforaphene Mitigates Periodontitis by NRF2‐Dependent Regulation of *P. gingivalis*‐Induced Inflammatory Response and Bone Homeostasis

**DOI:** 10.1002/fsn3.71319

**Published:** 2025-12-07

**Authors:** Hantao Yao, Kai Yu, Bulin Jiang, Yilin Liao, Yaoyu Zhao, Jingqiu Chen, Ting Li, Mengjie Yin, Yue Sheng, Wengwanyue Ye, Boxuan Zhao, Minquan Du, Yaoting Ji

**Affiliations:** ^1^ State Key Laboratory of Oral & Maxillofacial Reconstruction and Regeneration, Key Laboratory of Oral Biomedicine Ministry of Education, Hubei Key Laboratory of Stomatology, School & Hospital of Stomatology Wuhan University Wuhan China; ^2^ Stomatology Hospital, School of Stomatology Zhejiang University School of Medicine, Zhejiang Provincial Clinical Research Center for Oral Diseases, Zhejiang Key Laboratory of Oral Biomedical, Cancer Center of Zhejiang University, Engineering Research Center of Oral Biomaterials and Devices of Zhejiang Province Hangzhou China

**Keywords:** inflammation, NRF2, osteoclast differentiation, periodontitis, *P. gingivalis*, sulforaphene

## Abstract

Periodontitis is a prevalent chronic inflammatory disease closely associated with various systemic disorders. Conventional therapies, including mechanical debridement and antibiotics, are often insufficient to fully resolve inflammation or restore bone homeostasis. Sulforaphene (LFS) is abundant in radish seed oil and Lai Fu‐zi, a traditional Chinese herbal medicine and has been recognized for its anti‐inflammatory potential. Here, we show that LFS markedly attenuates the secretion of pro‐inflammatory cytokines (IL‐1β, IL‐6, and IL‐8) from human gingival fibroblasts and inhibits osteoclast differentiation even under IL‐1β‐induced inflammatory conditions, without compromising the osteogenic capacity of periodontal ligament cells (PDLC). Mechanistically, LFS activates the NRF2 pathway and its protective effect against alveolar bone loss was evident in *Nrf2* ^+/+^ mice but not *Nrf2* ^−/−^ mice. Notably, LFS was also found to inhibit the growth, virulence, and biofilm formation of *P. gingivalis* in vitro, and reduced its abundance within the subgingival microbiota in vivo. Together, these findings identify LFS as a potent NRF2‐dependent modulator, offering a promising therapeutic strategy for the prevention and treatment of periodontitis.

## Graphical abstract

Sulforaphene (LFS) directly inhibits *P. gingivalis* growth, biofilm formation, and virulence. It suppresses the inflammatory response in gingival fibroblasts and potently inhibits osteoclast differentiation, even under inflammatory conditions, without impairing osteogenesis. Critically, LFS activates the NRF2 pathway, and its protective effect against alveolar bone loss in experimental periodontitis is strictly dependent on NRF2, as demonstrated using *Nrf2* ^−/−^ mice.

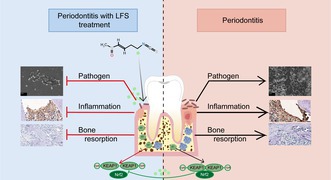

## 1 Introduction

Periodontitis is a chronic inflammatory disease characterized by the progressive destruction of periodontal supporting tissues, affecting an estimated 62% of dentate adults, with severe cases accounting for 23.6% (Hajishengallis et al. 2020; Trindade et al. 2023). Persistent periodontal inflammation can ultimately lead to tooth loss, severely compromising mastication, speech, and overall quality of life (Chen et al. 2021; Jiao et al. 2021). In recent years, accumulating evidence has also established a strong association between periodontitis and systemic inflammatory disorders such as atherosclerosis and Alzheimer's disease (Dominy et al. 2019; Tonetti et al. 2007, 2013), underscoring the importance of its prevention and management for both oral and systemic health.

Periodontitis is a destructive disease driven by inflammation of the periodontal tissues in response to toxins secreted by pathogenic bacteria such as *Porphyromonas gingivalis* (Socransky and Haffajee 2005). Under physiological conditions, osteoclasts and osteoblasts act in concert to maintain bone homeostasis; however, chronic inflammatory stimulation leads to excessive osteoclast differentiation and activation, resulting in connective tissue degradation and progressive alveolar bone resorption (Boyle et al. 2003; Teitelbaum and Ross 2003). Conventional treatments such as mechanical debridement and systemic antibiotics aim to reduce the bacterial load but often fail to adequately resolve the dysregulated host inflammatory response and aberrant osteoclast activity (Graziani et al. 2017; Slots 2017). Consequently, achieving predictable and complete periodontal regeneration using current technologies and materials remains a considerable challenge (Bao et al. 2016).

Nuclear factor erythroid 2–related factor 2 (NRF2) plays a central role in orchestrating cellular defense against oxidative stress. Under homeostatic conditions, NRF2 is sequestered in the cytoplasm by its repressor KEAP1 and targeted for ubiquitin‐mediated degradation. Upon activation, NRF2 dissociates from KEAP1, translocates to the nucleus, and promotes the transcription of a broad array of antioxidant and cytoprotective genes (Yamamoto et al. 2018). Studies using primary cells and established cell lines have demonstrated that NRF2 activation suppresses osteoclast differentiation, whereas its inhibition exerts the opposite effect (Han et al. 2022). Thus, pharmacological activation of NRF2 represents a promising therapeutic strategy to attenuate osteoclastic bone resorption and improve the outcomes of periodontal therapy (Malihe Arabpour et al. 2025).

A growing body of evidence has highlighted the therapeutic potential of traditional Chinese herbal extracts, which exhibit broad‐spectrum anti‐inflammatory activity through multitarget mechanisms with minimal adverse effects (Inagaki et al. 2021; Moghadam et al. 2020; Xia et al. 2023). Owing to their efficacy, such compounds have gained increasing acceptance in chronic inflammatory disease management. LFS, a natural isothiocyanate abundant in radish seed oil, is known for its potent anti‐inflammatory and cytoprotective properties in models of LPS‐induced bone erosion, neuroinflammation, gastritis, and colitis (Wang et al. 2018; Yang et al. 2020; Yao et al. 2023). In this study, we demonstrate, for the first time, LFS not only directly suppresses the growth and virulence of *Porphyromonas gingivalis* but also alleviates periodontal inflammation and alveolar bone loss in an NRF2‐dependent manner. Collectively, these results identify LFS as a multitarget NRF2 activator with significant potential as a novel therapeutic candidate for the prevention and treatment of periodontitis.

## 2 Materials and Methods

### 2.1 *P. gingivalis* and Culture Condition

The TSB culture medium for *P. gingivalis* (ATCC 33277) was prepared according to the following formulation: 30 g of trypticase soy broth (TSB, Becton, Dickinson and Company, USA), 5 g of yeast extract, 0.5 g of L‐cysteine hydrochloride, and 0.5 mg of hemin dissolved in 1000 mL of double‐distilled water. After sterilization by autoclaving at 121°C, the TSB culture medium was allowed to cool to room temperature. In a super‐clean workbench, 1 mg of vitamin K1 dissolved in anhydrous ethanol was added to the medium. 5% sheep blood and 15 mg/mL agar were added to obtain the blood agar plates.

The bacteria were maintained in a chamber (Anoxomat Mark, the Netherlands) at 37°C with anaerobic condition (10% H_2_, 10% CO_2_, and 80% N_2_). In the subsequent experiments, a bacterial concentration of 1.0 × 10^9^ CFUs/mL was achieved when the absorbance (OD) value reached 1.0 at 600 nm as measured by an ultraviolet spectrophotometer (SHIMADZU, Japan).

### 2.2 Enzyme‐Linked Immunosorbent Assay

Human gingival fibroblasts (HGFs) were cultured in minimum essential medium α (α‐MEM, Hyclone, USA) added with 1 μg/mL LPS from *P. gingivalis* for 24 h to secret inflammatory cytokines. And LFS (0, 1 and 5 μM) intervenes in this process of inflammation induction. The working concentrations of LFS (1 and 5 μM) used in this study were selected based on our previous cytotoxicity assays, which demonstrated no significant cytotoxicity to relevant cell lines at concentrations up to 6.25 μM (Yao et al. 2023). After the induction, the cell supernatants were collected and measured according to the instructions of enzyme‐linked immunosorbent assay (ELISA) kits (NeoBioscience, China) for concentrations of inflammatory factors such as IL‐1β (Catalogue number: EHC002b, NeoBioscience, China), IL‐6 (Catalogue number: EHC007, NeoBioscience, China) and IL‐8 (Catalogue number: EHC008, NeoBioscience, China).

### 2.3 Osteoclastogenesis Induction

Eight‐week‐old C57BL/6 mice were used to obtain bone marrow cells by flushing the femur and tibia. The collected cells were suspended in α‐MEM plus 10% FBS (α‐10) (Biological Industries, China) and cultured overnight in a 10 cm culture dish. After centrifugation, the supernatant was collected, and the cells were resuspended in culture medium supplemented with 30 ng/mL macrophage‐colony‐stimulating factor (M‐CSF, Novoprotein, China). The cells were then cultured in a petri dish and allowed to proliferate for 2–3 days. Bone marrow macrophages (BMMs) were identified by their adherence to the culture dish. Subsequently, the cells were detached using trypsin and seeded onto culture dishes at a density of 2.5 × 10^4^ cells/cm^2^. Once the cells adhered to the dish, they were induced with α‐10 medium containing 30 ng/mL M‐CSF and 100 ng/mL receptor activator of nuclear factor‐kB ligand (RANKL). The culture medium was replaced every 2 days, and after 4–6 days, visible osteoclast formation was observed. To simulate an inflammatory environment, IL‐1β was used as a treatment. Various concentrations of IL‐1β (0, 10, 20, 40 ng/mL) were added to the culture medium during the induction phase. To evaluate the effects of LFS, different concentrations of LFS (0, 1, and 5 μM) were added to the culture medium under the conditions mentioned above.

### 2.4 Tartrate‐Resistant Acid Phosphatase Staining and Fibrous Actin Rings Immunofluorescence

On days 4–6 of osteoclast induction, upon the appearance of evident osteoclasts, the cells in the wells were fixed with 4% paraformaldehyde for 15 min. Following fixation, the cells underwent three washes with PBS. Subsequently, the cells were subjected to staining using a tartrate‐resistant acid phosphatase (TRAP) staining kit (Sigma‐Aldrich, USA) as the provided instructions. For fluorescence staining of actin, the fixed cells were permeabilized with 0.1% Triton‐X and blocked with 4% bovine serum albumin. TRITC‐labeled phalloidin (Yeasen, China) was applied to the cells, allowing for specific binding to actin filaments. The staining process was carried out in a dark environment for 30 min. Finally, the cells were counterstained with DAPI (Beyotime, China) to visualize the cell nuclei. The stained cells were examined and images were captured using a fluorescence microscope (Leica, Germany). The number of nuclei per osteoclast and the osteoclast area were quantified with ImageJ software.

### 2.5 Periodontal Ligament Cell Culture and Osteogenic Induction

PDLCs were obtained as reported previously (Xiao et al. 2023). Briefly, periodontal ligament tissue was obtained from extracted teeth and minced or enzymatically digested to release the cells at 37°C for an hour. After centrifugation, the cells were resuspended and cultured in medium with FBS. Passages 3 to 7 of the cells were used in the experiment. For osteogenic induction, specific substances, including 10 nM dexamethasone (Sigma‐Aldrich, USA), 50 μg/mL ascorbic acid (Sigma‐Aldrich, USA), and 10 mM β‐glycerophosphate (Sigma‐Aldrich, USA), were added to the culture medium. The culture medium was replaced every 2 days. After 7 days of culturing, ALP staining and activity were determined by BCIP/NBT ALP Color Development Kit (Beyotime, China) according the instructions. ALP activity were quantified with ImageJ software.

### 2.6 Total RNA Isolation and Quantitative Reverse Transcription‐Polymerase Chain Reaction Analysis

The cells were subjected to RNA extraction using Trizol reagents (Takara, Japan) to obtain total RNA. RNA concentration and purity were quantified on a Nanodrop 2000 spectrophotometer (ThermoFisher, USA). The PrimeScriptTM Reverse Transcription Reagent Kit (Vazyme, China) was employed to reverse transcribe 1 μg of RNA into complementary DNA (cDNA). Quantitative real‐time PCR (qPCR) was conducted on an Applied Biosystems QuantStudio 6 instrument (Thermo, USA) using SYBR qPCR Master Mix (Vazyme, China). The thermal cycling protocol, as per the manufacturer's instructions, comprised an initial denaturation at 95°C for 30 s, followed by 40 cycles of denaturation at 95°C for 10 s and annealing/extension at 60°C for 30 s. The gene expression ratios were determined using the 2^−ΔΔ*Ct*^ method, with normalization to the reference gene Rn18s (18s). The specific primer sequences used for qPCR analysis can be found in Table 1.

**TABLE 1:** Specific primers used for qPCR.

Genes	Forward primer	Reverse primer
*fimA*	TCTTGTTGGGACTTGCTGCTCTTG	CGCTGATGGTGGCATTACCTTCTG
*kgp*	ACCTACACTCAAGGAGGAGCCAAC	GGACCTTCGCCTTCACCTGTTATC
*P. gingivalis 16S*	GGTGCGTAGGTTGTTCGGTAAGTC	CTGCCGCCGCTGAACTCAAG
*IL‐1β*	ATGATGGCTTATTACAGTGGCAA	GTCGGAGATTCGTAGCTGGA
*IL‐6*	CACTGGTCTTTTGGAGTTTGAG	GGACTTTTGTACTCATCTGCAC
*IL‐8*	ACTGAGAGTGATTGAGAGTGGAC	AACCCTCTGCACCCAGTTTTC
*TNF‐α*	GCCCATGTTGTAGCAAACCC	TGAGGTACAGGCCCTCTGAT
*COX2*	GCAAATTGCTGGCAGGGTTG	GCTCTGGTCAATGGAAGCCT
*ALPL*	CTATCCTGGCTCCGTGCTCC	TTAACTGATGTTCCAATCCTGCG
*RUNX2*	CGCCTCACAAACAACCACAG	ACTGCTTGCAGCCTTAAATGAC
*OPN*	ACAAATACCCAGATGCTGTGGC	ACTTGGAAGGGTCTGTGGGG
*OCN*	CATGAGAGCCCTCACACTCC	TGCTTGGACACAAAGGCTGC
*Traf6*	GATCCAGGGCTACGATGTGG	CTTGTGCCCTGCATCCCTTA
*Ctsk*	TAGCACCCTTAGTCTTCCGC	CTTGAACACCCACATCCTGC
*Mmp9*	CAGCCGACTTTTGTGGTCTTC	CGGTACAAGTATGCCTCTGCCA
*Trap*	GACCCACCGCCAAGATGGAT	CACGGTTCTGGCGATCTCTT
*Dc‐stamp*	GGAACCTAAGCGGAACTTAGAC	CACATAAGTCCCCCAAAGCCT
*Nfatc1*	TGGAGAAGCAGAGCACAGAC	GCGGAAAGGTGGTATCTCAA
*Calcr*	GCAGGCACTGCTAAGGAGA	GGTGTTCTCAGGAACGCAGA
*18 s*	AGTCCCTGCCCTTTGTACACA	CGATCCGAGGGCCTCACTA
*Gapdh*	GGTTGTCTCCTGCGACTTCA	TGGTCCAGGGTTTCTTACTCC

### 2.7 Western Blot

RIPA lysis buffer solution (Beyotime, China) supplemented with phosphatase inhibitor (Roche, Germany) and protease inhibitor (Beyotime, China) was used to collect cellular protein and the BCA protein assay kit (Beyotime, China) was employed to determine the protein concentration. Protein samples were separated by electrophoresis on 10% SDS‐polyacrylamide gels under the following conditions: 80 V for 30 min, followed by 110 V for 1 h. Subsequently, the separated proteins were electrophoretically transferred onto a 0.45 μm PVDF membrane, which had been pre‐activated with methanol, at a constant current of 200 mA for 100 min. Following blocked with blocking buffer (Beyotime, China), the membranes were incubated overnight at 4°C with primary antibodies specific to SP7 (Catalogue number: ab209484, Abcam, USA), OCN (Catalogue number: b0822, Santa Cruz Biotechnology, USA), NFATC1 (Catalogue number: d0522, Santa Cruz Biotechnology, USA), IL‐1β (Catalogue number: A19635, ABclonal Technology, China), IL‐6 (Catalogue number: A0286, ABclonal Technology, China), CTSK (Catalogue number: PB9856, Bosterbio, China), MMP9 (Catalogue number: ab76003, Abcam, USA), ATP6V0D2 (Catalogue number: 333641‐ap, Proteintech, China), TRAP (Catalogue number: ab133238, Abcam, USA), and β‐TUBULIN (Catalogue number: M20045F, Abmart, China) at 4°C overnight. Then the membranes were further incubated with HRP‐conjugated secondary antibodies (Abbkine, China) at a dilution of 1:10,000. Finally, an ECL system (Millipore, USA) was employed to detect the specific protein bands of interest.

### 2.8 Molecular Docking Simulation

The three‐dimensional structure of LFS (PubChem CID: 6433206) was retrieved from the NCBI PubChem database, while the crystal structure of KEAP1 was obtained from the Protein Data Bank (PDB ID: 4L7B). Molecular docking of LFS with KEAP1 was performed using AutoDock Vina. The resulting docking poses were visualized, and representative images were generated with PyMOL.

### 2.9 RNA Sequencing and Analysis

Raw264.7 were induced into osteoclast using RANKL with or without LFS intervention (*n* = 3 biological replicates per group). RNA was extracted as described earlier and transcriptome analysis was performed on the Illumina HiSeq2500 with paired‐end sequencing by Genesky Biotechnologies Inc. (Shanghai, China). Further bioinformatics data analysis and visualization were executed on an online platform (www.bioinformatics.com.cn).

### 2.10 Mouse Periodontitis Model Protocols

The animal experiments conducted in this study were approved and supervised by the Institutional Animal Care and Use Committee of Wuhan University, China (permission number: S07920120I). Part one: A total of 15 eight‐week‐old male C57BL/6 mice were randomly assigned to one of three groups (*n* = 5 per group) according to random number table: (1) sham surgery (Sham); (2) periodontitis group (PD), and Periodontitis treatment with LFS (PD + LFS). Part two: To assess the role of *Nrf2*, 4 eight‐week‐old male C57BL/6 mice (wild‐type) and 12 *Nrf2* ^*−/−*^ mice of same background and age were used. These 16 mice were randomly divided into four groups (*n* = 4 per group): (1) *Nrf2* ^*+/+*^ wild‐type (WT); (2) the *Nrf2* ^*−/−*^ control (*Nrf2* ^*−/−*^ control); (3) *Nrf2* ^*−/−*^ periodontitis (*Nrf2* ^*−/−*^ PD) and (4) *Nrf2* ^*−/−*^ periodontitis treated with LFS (*Nrf2* ^*−/−*^ PD + LFS). A mouse model of periodontitis was established by bilaterally ligating the maxillary second molars with 5–0 silk sutures, while mice in the sham group underwent a sham surgery without ligation. The PD + LFS group received daily intraperitoneal injections of 20 mg/kg/day LFS (dissolved in saline) with an injection volume of 5 mL/kg body weight. Meanwhile the SHAM and PD group received equal volumes of saline in the following week. Significant periodontal bone loss could be visible in PD group after a week. Finally, mice in the three group were euthanized by CO_2_ inhalation, was inoculated onto a blood agar plate, followed by collection of alveolar bone and visceral tissues for subsequent analysis. Before euthanasia, gingival crevicular fluid was sampled by blotting the gingival sulcus with a #20 absorbent paper point for 5 s. The paper point was eluted in sterile PBS at 4°C overnight. Following euthanasia, alveolar bone and visceral tissues were harvested. For bacterial analysis, 5 μL of the eluted liquid was plated on blood agar and cultured for 1 week.

### 2.11 Histological Analysis

Alveolar bones were fixed with 4% paraformaldehyde for 2 days and underwent a decalcification process with 10% ethylene diamine tetraacetic acid (EDTA) for 1 month. After dehydration and paraffin embedding, the tissues were sectioned into 5 μm slices. Subsequent histological evaluations, including hematoxylin–eosin (HE) staining, TRAP staining and immunohistochemistry (IHC) were performed according to previous studies (Yao et al. 2023). The primary antibodies used for IHC include the following: IL‐1β (Catalogue number: GB300002, Servicebio, China), IL‐6 (Catalogue number: GB300007, Servicebio, China), and TNF‐α (Catalogue number: a11534, ABclonal Technology, China).

### 2.12 Stereomicroscopy and Micro‐CT Analysis

To facilitate clear observation of periodontal bone loss, soft tissues were meticulously removed to fully expose the alveolar bone. The maxillae were stained with 0.3% methylene blue and imaged under a stereomicroscope (Zeiss Axio Observer, Germany). The micro‐CT system (Skyscan 1276, Bruker, Germany) was used to scan the samples. The Skyscan Ctan software was carried out to analyze the region of interest (ROI), which was referred as the region of bone located between the intermediate line of the first molar and second molar, extending to the intermediate line of the second molar and third molar. Three‐dimensional reconstructions were generated with Skyscan CTvox software. This ROI was analyzed using Skyscan CTAn software to calculate bone morphometric parameters. The distance from the cemento‐enamel junction to the alveolar bone crest (CEJ‐ABC) was measured to quantify bone loss.

### 2.13 Effects of LFS on *P. gingivalis* Growth

Sulforaphene was purchased from De‐Si‐Te Company (DL0137, Purity: ≥ 98%). *P. gingivalis* with an initial concentration of 1 × 10^8^ CFU/mL was treated with various concentrations of LFS (0, 1, 5 and 10 μM) in 96‐well plates and incubated in TSB medium for 24 h. Following the incubation period, the optical density (OD) values of the samples were measured at 600 nm using a microplate reader (BioTek Instruments, USA).

### 2.14 Crystal Violet Assay

*P.gingivalis* biofilms were treated with LFS (0, 1 and 5 μM) in 96‐well plates for 48 h and the plates were washed with sterile water for three times. The adherent biofilms were fixed and stained with 0.1% crystal violet (Shanghai Hushi, China) for 15 min. After staining, the dye solution was discarded, and the wells were thoroughly rinsed three times with sterile water to remove any unbound dye. Then the representative images were taken and the crystal violet was dissolved with an appropriate amount of 30% acetic acid which was measured at 562 nm by a microplate reader for OD values.

### 2.15 Confocal Laser Scanning Microscopy Analysis

*P. gingivalis* was cultured in 24‐well plates and treated with LFS (0, 1, and 5 μM) on coverslips. After 72 h, *P. gingivalis* were washed twice by saline to remove non‐adherent cells. Bacterial viability was assessed using the LIVE/DEAD BacLight Bacterial Viability Kit (ThermoFisher, USA), according to the manufacturer's instructions. This kit contains a mixture of SYTO 9 and propidium iodide. After incubation in the dark for 15 min, the samples were imaged. The stained samples were observed under a confocal laser scanning microscope (CLSM; Leica Biosystems, Germany) using a 488 nm laser for excitation, with emission filters set to 500–550 nm for SYTO 9 (live cells, green) and 600–650 nm for propidium iodide (dead cells, red), and three‐dimensional images were reconstructed using the Leica Application Suite X (LAS X) software.

### 2.16 Scanning Electron Microscopy Analysis

The samples were prepared according to the above method. Following overnight fixation, they were dehydrated through a graded ethanol series and subjected to critical point drying to remove residual moisture. The samples were sputter‐coated with gold for 120 s. The prepared samples were placed on the scanning electron microscope table, and appropriate electron beam conditions were set to achieve high‐resolution imaging. The morphology and structure of the bacterial biofilms were examined and recorded using a scanning electron microscope (SEM; Sigma, Zeiss AG, Germany).

### 2.17 Statistical Analysis

All experiments were conducted at least three times and GraphPad Prism Version 8.0 software was carried out to perform data analysis. The results are visualized as the mean ± standard deviation (SD). The unpaired t‐test and one‐way ANOVA followed by Tukey's post hoc test were used for comparisons between two groups and multiple groups, respectively. Differences were deemed statistically significant at *p < 0.05.

## 3 Results

### 3.1 LFS Down‐Regulates the Inflammatory Response of Gingival Fibroblasts

**FIGURE 1:**
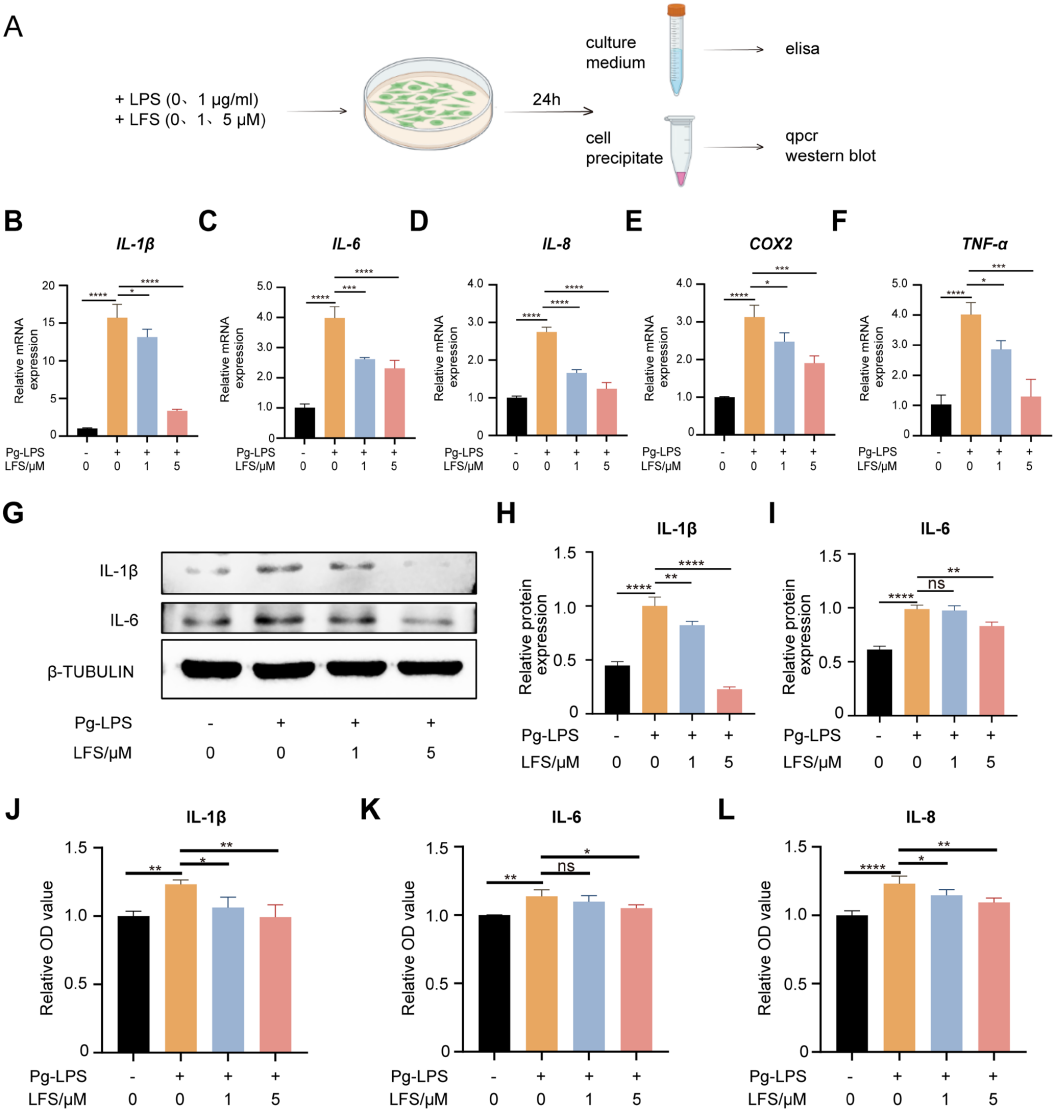
LFS down‐regulates the inflammatory response of gingival epithelial cells. (A) Schematic diagram of *P. gingivalis*‐LPS induced HGF inflammation and intervention of LFS. (B–F) Relative mRNA expression levels of *IL‐1β, IL‐6*, *IL‐8*, *TNF‐α* and *Cox2* of HGF with LFS intervention by qPCR. (G) Representative images of western blot of IL‐1β and IL‐6 of HGF in the presence of LFS (0, 1, and 5 μM). (H, I) The protein expression levels ratio of IL‐1β/β‐TUBULIN and IL‐6/β‐TUBULIN of HGF. (J–L) Relative concentrations of IL‐1β，IL‐6 and IL‐8 of HGF with LFS (0, 1 and 5 μM) measured by elisa. Data are presented as means ± SD of 3 independent experiments; *p < 0.05, **p < 0.01, ***p < 0.001, and ****p < 0.0001.

In this study, the impact of LFS on the expression of pro‐inflammatory cytokines were investigated firstly (Figure 1A). qPCR analysis revealed that LFS effectively suppressed the mRNA expression of *IL‐1β*, *IL‐6*, *IL‐8*, *TNF‐α*, and *COX2* (Figure 1B–F). Treatment with LFS at 1 and 5 μM significantly attenuated this upregulation in a concentration‐dependent manner. Specifically, the 5 μM LFS treatment reduced the mRNA levels of these cytokines to levels comparable to, or even lower than, the unstimulated control.

Furthermore, western blot assays demonstrated significant inhibition of IL‐1β and IL‐6 protein expression upon LFS treatment (Figure 1G–I). Quantitative analysis showed that both 1 and 5 μM LFS significantly decreased the protein ratios of IL‐1β/β‐TUBULIN and IL‐6/β‐TUBULIN compared to the LPS‐only group.

The release of these inflammatory factors contributes to the establishment of a local inflammatory microenvironment. To further elucidate the effect of LFS, we conducted Elisa assays to explore the release of IL‐1β, IL‐6, and IL‐8. Consistently, the results confirmed that LFS markedly impeded the release of these inflammatory factors (Figure 1J–L). Notably, the 5 μM LFS treatment reduced the secretion of IL‐1β, IL‐6, and IL‐8 to levels that were not significantly different from the baseline observed in the unstimulated control group.

### 3.2 LFS Significantly Impedes Osteoclast Differentiation Without Affecting Osteogenesis

The preservation of bone mass relies on the intricate interplay between osteoblasts and osteoclasts. In this study, we sought to investigate the effects of LFS on osteoclastic differentiation of BMDMs and osteoblast differentiation of PDLCs.

**FIGURE 2:**
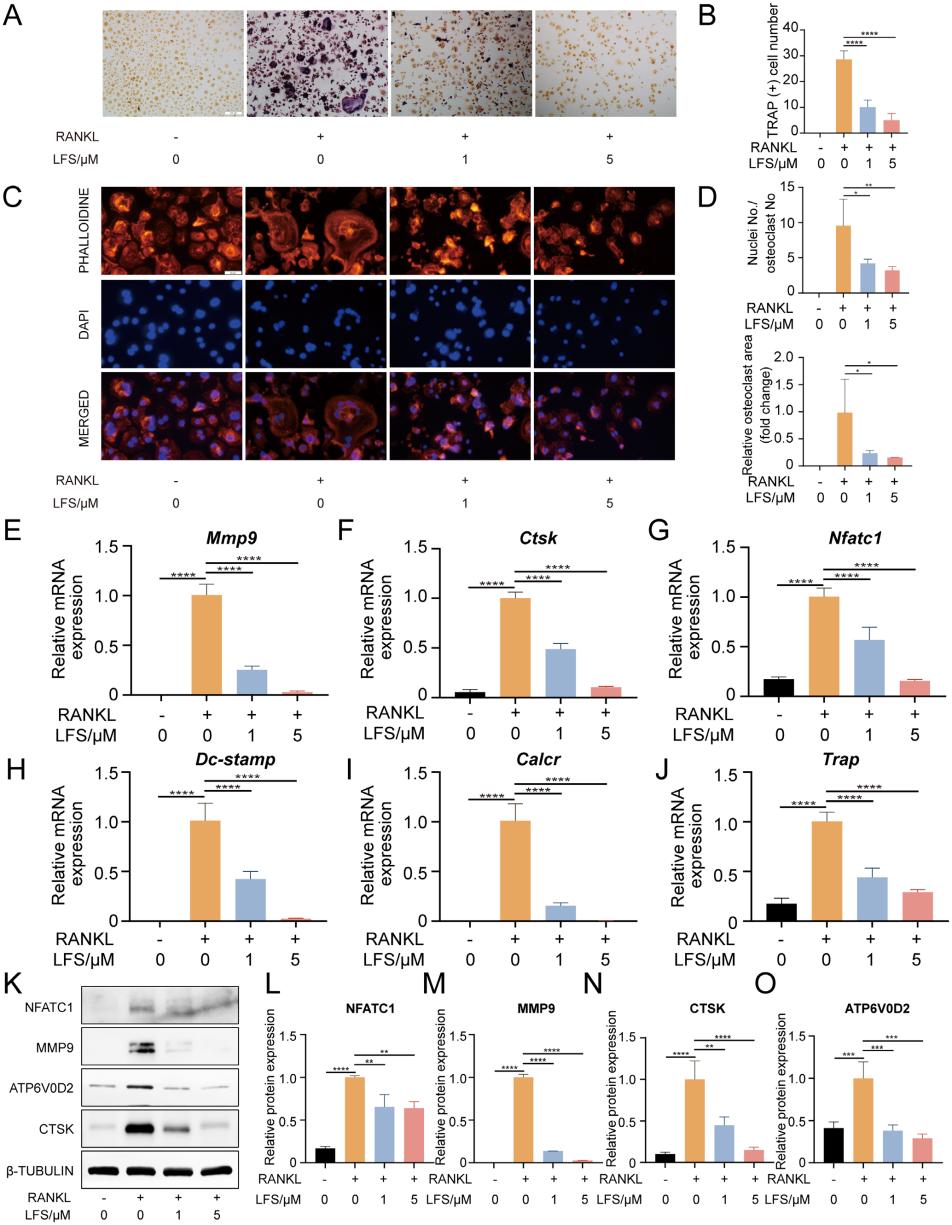
LFS impedes osteoclast differentiation of BMDM in vitro. (A, B) Representative images of TRAP staining of RANKL‐induced BMDM with different concentration of LFS (0, 1, and 5 μM) and quantitative analysis. Scale bar = 100 μm. (C, D) Representative images of F‐actin ring staining (phalloidin, red) and nuclei (DAPI, blue) of BMDMs, and subsequent quantitative analysis of osteoclast area and the number of nuclei per osteoclast. Scale bar = 50 μm. (E–J) Relative mRNA expressions of osteoclast differentiation related genes of BMDM with LFS (0, 1, and 5 μM) by qPCR. (K–O) Representative western blot images of NFATC1, MMP9, CTSK, ATP6V0D2 and β‐TUBULIN of BMDM with LFS and respective quantitative analysis. Data are presented as means ± SD of 3 independent experiments; *p < 0.05, **p < 0.01, ***p < 0.001, and ****p < 0.0001.

Mature osteoclasts were induced through M‐CSF and RANKL stimulation to achieve complete differentiation. TRAP staining revealed extensive formation of multinucleated TRAP‐positive osteoclasts in the RANKL‐induced control group (Figure 2A,B). This differentiation was significantly inhibited by LFS. The number of TRAP‐positive osteoclasts was reduced by approximately 65% and 82% in the 1 and 5 μM LFS treatment groups, respectively, compared to the RANKL‐only group.

The inhibitory effect of LFS on osteoclast function was further visualized by fibrous actin (F‐actin) ring staining (Figure 2C,D). The podosome belt is the signature structure responsible for osteoclast function. While large, well‐defined F‐actin rings were abundant in the control group, LFS treatment resulted in a significant, concentration‐dependent reduction in both the number and size of these structures, indicating impaired cytoskeletal organization essential for bone resorption. Additionally, qPCR analysis demonstrated substantial downregulation of multiple osteoclastic differentiation marker genes, including *Traf6*, *Nfatc1*, *Ctsk*, *Mmp9*, *Trap*, and *Dc‐stamp* (Figure 2E–J). The addition of LFS (1 and 5 μM) during RANKL induction led to a substantial and statistically significant downregulation of all these marker genes compared to the RANKL‐only group. Western blot results further underscored LFS's inhibitory influence on key osteoclast‐related proteins, including NFATC1, MMP9, CTSK, and ATP6V0D2, which were otherwise upregulated under RANKL stimulation (Figure 2K–O).

**FIGURE 3:**
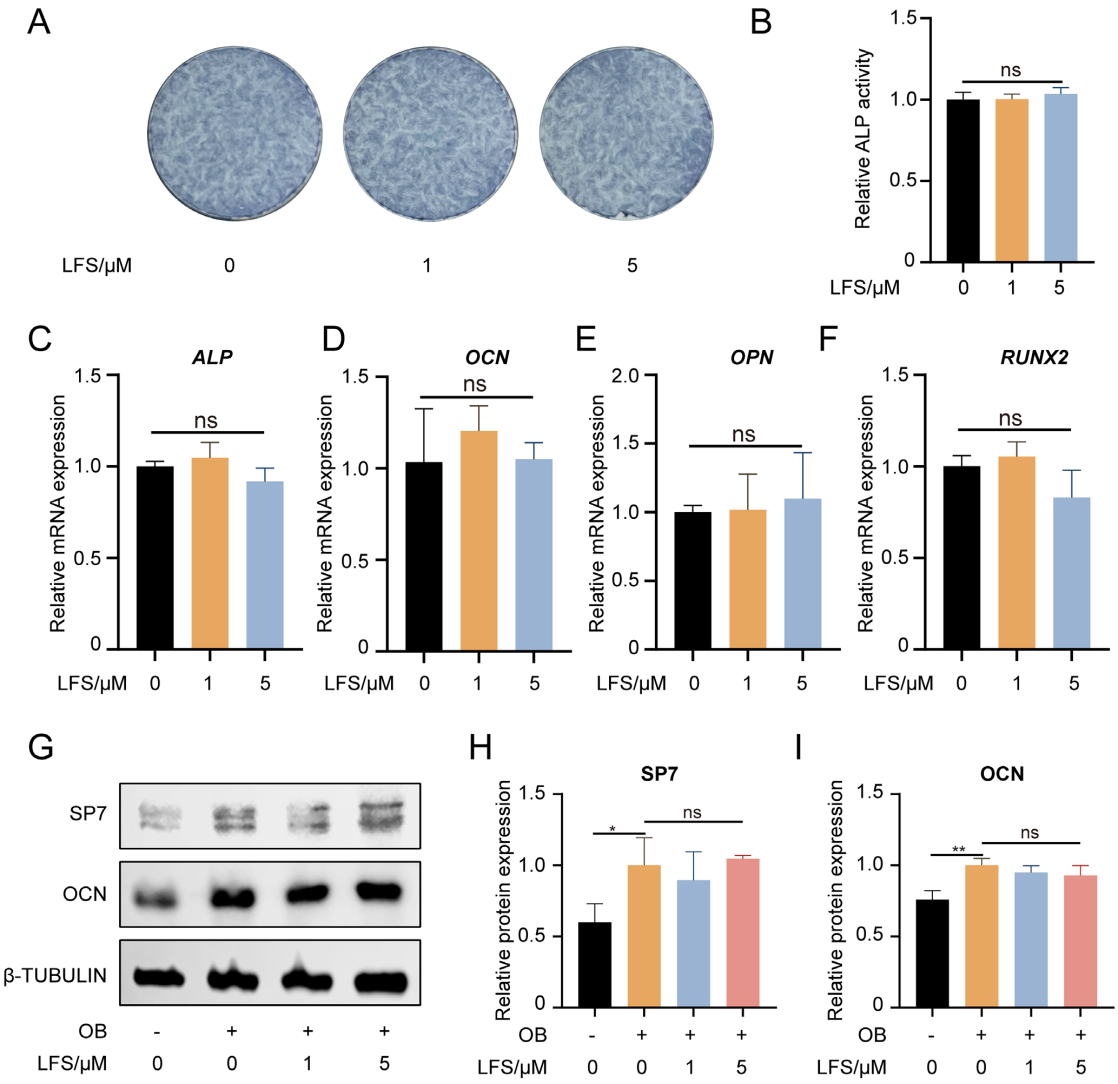
LFS does not inhibit osteogenic differentiation of PDLC. (A) Representative images of ALP staining of PDLC with different dose of LFS after osteogenic induction. Scale bar = 100 μm. (B) Relative ALP activity of PDLC with different dose of LFS after osteogenic induction. (C–F) Relative mRNA expressions of *ALP*, *OCN*, *OPN* and *RUNX2* of PDLC with different dose of LFS after osteogenic induction. Scale bar = 100 μm. (G–I) Representative western blot images of SP7, OCN and β‐TUBULIN of PDLC and respective quantitative analysis. Data are presented as means ± SD of 3 independent experiments; *p < 0.05 and **p < 0.01.

Given that some anti‐osteoclastogenic agents inadvertently suppress bone formation, we investigated the impact of LFS on the osteogenic differentiation of PDLCs (Kong et al. 2023; Iwata et al. 2006; Manzano‐Moreno et al. 2015). It is necessary to find interventions that can selectively regulate bone remodeling without compromising osteogenesis. To address this, we assessed the impact of LFS (0, 1 and 5 μM) on PDLCs osteogenesis. Alkaline phosphatase (ALP) staining and activity revealed that LFS had no significant effect on PDLCs osteogenic potential (Figure 3A,B). At the mRNA level, LFS at 1 μM appeared to enhance the expression of osteogenic genes, although statistical significance was not observed (Figure 3C–F). Of paramount importance, western blot experiments indicated that the protein levels of SP7 and OCN, vital osteoblast markers, were not hindered by LFS treatment (Figure 3G–I). These results collectively indicate that LFS does not impair the osteogenic potential of PDLCs.

### 3.3 LFS Inhibits Osteoclast Differentiation in an IL‐1β‐Induced Inflammatory Microenvironment

**FIGURE 4:**
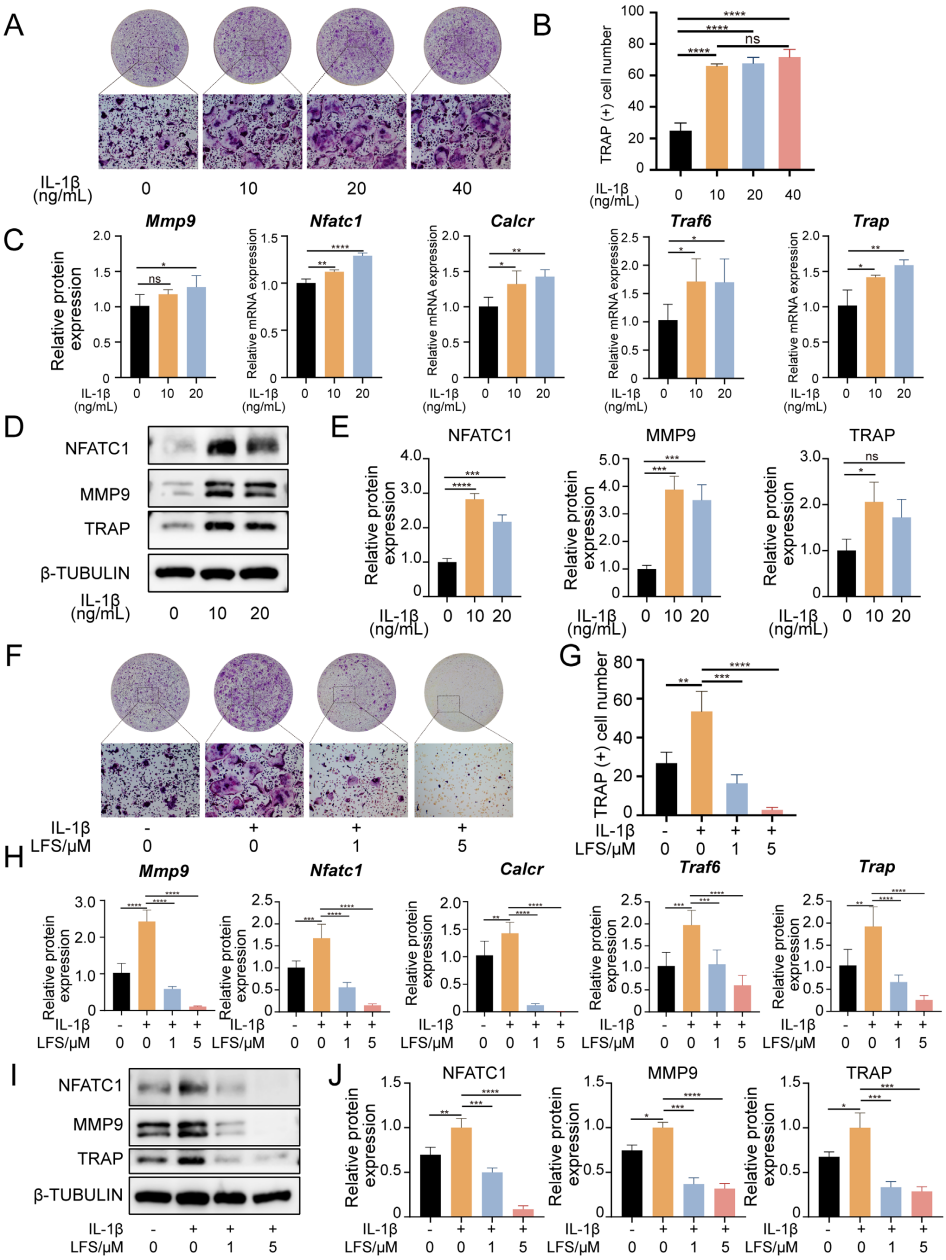
LFS takes an inhibitory effect on osteoclast differentiation in the presence of IL‐1β. (A, B) Representative TRAP staining images of BMDM with RANKL induction and different concentration of IL‐1β (0, 10, 20 and 40 ng/mL), and quantitative analysis of TRAP (+) cell number. Scale bar = 100 μm. (C) Relative mRNA expressions of osteoclast differentiation related genes of BMDM with different concentration of IL‐1β (0, 10 and 20 ng/mL) by qPCR. (D, E) Representative western blot images of NFATC1, MMP9, TRAP and β‐TUBULIN of BMDM with RANKL induction and different concentration of IL‐1β (0, 10 and 20 ng/mL), and subsequent quantitative analysis. (F, G) Representative TRAP staining images of RANKL‐induced BMDM with different concentration of IL‐1β (0 and 10 ng/mL) or LFS (0, 1 and 5 μM), and quantitative analysis of TRAP (+) cell number. Scale bar = 100 μm. (H) Relative mRNA expressions of osteoclast differentiation related genes of BMDM with different concentration of IL‐1β (0 and 10 ng/mL) or LFS (0, 1 and 5 μM) by qPCR. (I, J) Representative western blot images of NFATC1, MMP9, TRAP and β‐TUBULIN of BMDM with RANKL induction and different concentration of IL‐1β (0 and 10 ng/mL) or LFS (0, 1 and 5 μM), and subsequent quantitative analysis.

Since IL‐1β is a pivotal inflammatory cytokine in periodontitis, we first established its effect on osteoclastogenesis (Cheng et al. 2020). TRAP staining showed that the addition of IL‐1β (10–40 ng/mL) to RANKL‐stimulated BMDMs significantly enhanced osteoclast formation compared to RANKL alone (Figure 4A,B). The promoting effect was maximal at 10 ng/mL, with no significant further increase at higher concentrations. Accordingly, qPCR and western blot analyses confirmed that 10 ng/mL IL‐1β significantly amplified the expression of osteoclast marker genes and proteins (NFATC1, MMP9, TRAP) (Figure 4C–E). Therefore, 10 ng/mL IL‐1β was selected to simulate the inflammatory microenvironment in subsequent experiments.

Subsequently, we explored LFS's effects on osteoclasts under an inflammatory milieu simulated by 10 ng/mL IL‐1β. Encouragingly, even in the presence of IL‐1β, LFS consistently exhibited a significant inhibitory impact on osteoclast differentiation (Figure 4F,G). The number of osteoclasts in the RANKL+IL‐1β + 5 μM LFS group was comparable to that in the group treated with RANKL alone. Furthermore, relevant indicators were consistently downregulated at both mRNA and protein levels (Figure 4H–J). The mRNA expression of Nfatc1, Ctsk, and Mmp9, which was synergistically upregulated by RANKL and IL‐1β, was significantly downregulated by LFS co‐treatment (Figure 4H). Western blot analysis yielded congruent results, showing that LFS reduced the protein levels of NFATC1, MMP9, and TRAP even in the presence of the pro‐osteoclastogenic inflammatory cue IL‐1β (Figure 4I,J).

These data unequivocally demonstrate that LFS retains its potent anti‐osteoclastogenic activity under inflammatory conditions mimicking periodontitis.

### 3.4 LFS Mitigates Periodontitis Bone Loss by Inhibiting Osteoclast Differentiation and Inflammatory Cytokine Expression In Vivo

**FIGURE 5:**
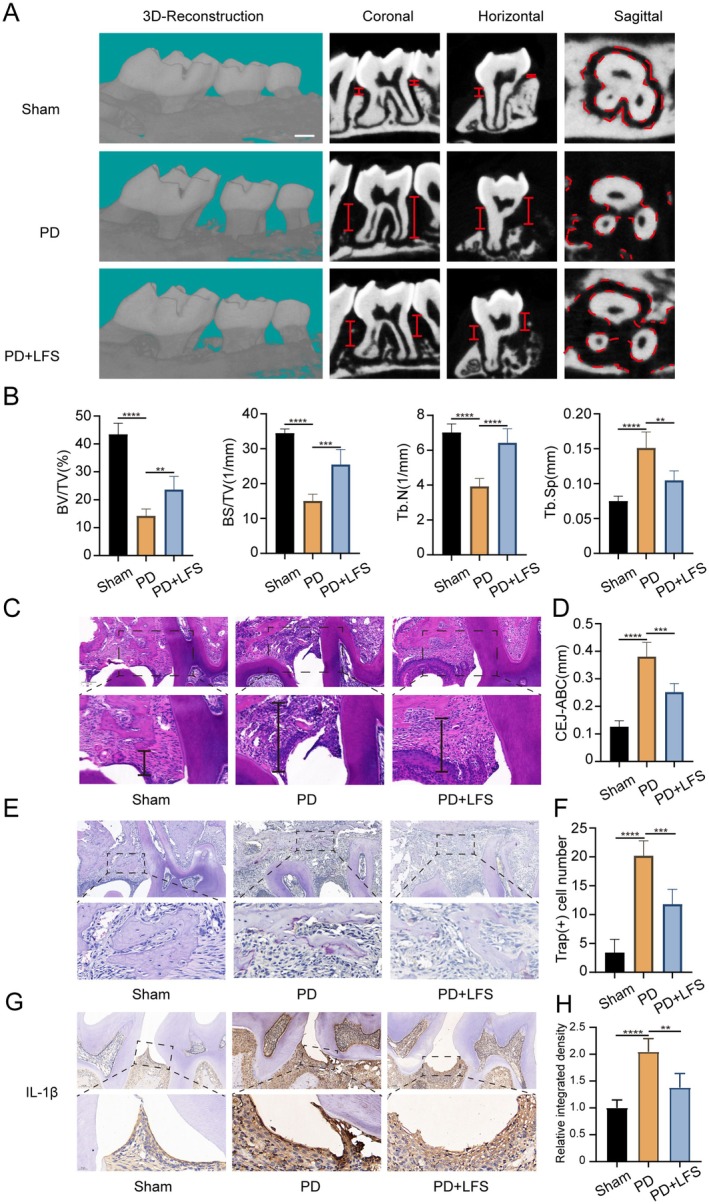
LFS mitigates alveolar bone loss and osteoclast differentiation in vivo. (A, B) Representative micro‐CT images in different groups and quantitative Analysis of bone volume per tissue volume (BV/TV) percentage, trabecular thickness (Tb.Th), trabecular number (Tb.N), and trabecular spacing (Tb.Sp) in different groups. (C, D) Representative HE staining images and measurements of the distances from the cemento‐enamel junction to the alveolar bone crest in different groups. Scale bar = 100 μm. (E, F) Representative TRAP staining images and quantitative analysis of osteoclast number in different groups in vivo. Scale bar = 100 μm. (G, H) Representative immunohistochemical images for the distribution of IL‐1β in vivo, and respective quantitative analysis of relative mean density of IL‐1β in each group. Scale bar = 100 μm. *n* = 5 per group; **p < 0.01, ***p < 0.001, and ****p < 0.0001.

To evaluate the therapeutic potential of LFS in vivo, we established a mouse model of periodontitis using silk ligature around the upper second molar, followed by daily intraperitoneal injection of LFS. Micro‐CT analysis revealed substantial alveolar bone loss around the second molar in the periodontitis (PD) group compared to the sham‐operated group (Figure 5A,B). Quantitative analysis showed a significant decrease in Bone Volume/Total Volume (BV/TV) and Trabecular Number (Tb.N), alongside a significant increase in Trabecular Spacing (Tb.Sp) in the PD group. Crucially, daily treatment with LFS (PD + LFS group) largely prevented this bone destruction, as evidenced by a significant recovery in BV/TV and Tb.N, and a reduction in Tb.Sp towards the levels observed in the sham group.

Consistent with the micro‐CT data, histological examination with H&E staining showed a notable reduction in alveolar bone height in the PD group. The distance from the cemento‐enamel junction to the alveolar bone crest (CEJ‐ABC) was significantly greater in the PD group than in the sham group. This ligature‐induced bone loss was significantly ameliorated in the PD + LFS group, where the CEJ‐ABC distance was markedly shorter than in the PD group (Figure 5C,D).

Additionally, TRAP staining demonstrated a significant increase in the number of TRAP‐positive osteoclasts lining the alveolar bone surface was observed in the PD group compared to the sham group. In contrast, the PD + LFS group exhibited a significantly lower number of osteoclasts, indicating that LFS inhibits osteoclast formation in vivo (Figure 5E,F).

Furthermore, immunohistochemical staining for IL‐1β revealed a strong inflammatory response in the periodontal tissues of the PD group (Figure 5G,H). The relative mean density of IL‐1β was significantly higher in the PD group than in the sham controls. Treatment with LFS significantly reduced the level of IL‐1β, bringing it closer to the baseline observed in the sham group. These in vivo findings collectively demonstrate that LFS alleviates ligature‐induced periodontitis by dampening the inflammatory response and suppressing osteoclast‐mediated bone resorption.

### 3.5 Inhibition of *Nrf2* Impairs the Protective Effect of LFS for Periodontitis

**FIGURE 6:**
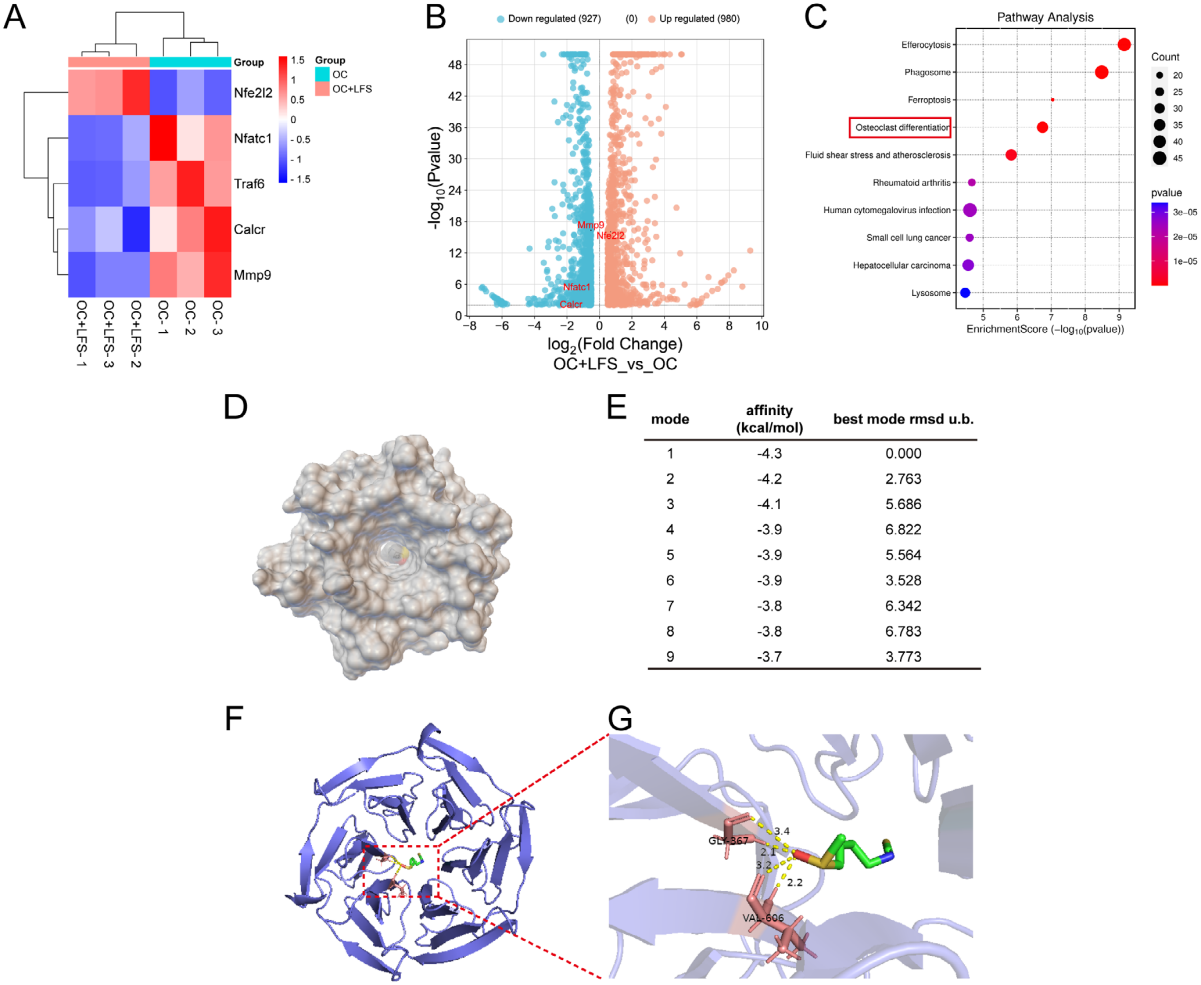
RNA‐Seq and molecular docking simulation reveal LFS impedes osteoclast differentiation via influencing the expression of *Nrf2*. (A) The expression of *Nrf2* and osteoclastogenesis‐related genes in OC and OC + LFS groups were negatively correlated showed by heat map (*n* = 3). (B) The related genes between the OC group and the OC + LFS group were marked by the volcano plot. (C) Further gene set enrichment analysis performed with the GO and KEGG databases demonstrated osteoclast differentiation pathway were significantly enriched. (D, E) Schematic diagram of the binding between LFS and KEAPI, and molecular docking simulation predicts the binding mode and binding energy of LFS‐KEAP1. (F, G) Molecular docking simulation predicted the binding sites of LFS and KEAP1.

In order to delve deeper into the mechanism of LFS, RNA sequencing and bioinformatics analyses were carried out on Raw264.7 cells stimulated by RANKL, in the presence or absence of LFS treatment. The heat map and volcano plot indicated that the expression of *Nfe2l2* (encoding NRF2) was upregulated, while a set of genes associated with osteoclast differentiation was downregulated in the LFS‐treated group compared to the RANKL‐induced control group (Figure 6A,B). Genes with adjusted *p*‐value < 0.01 and absolute log fold change > 0.5 were deemed differentially expressed genes (DEGs). Gene set enrichment analysis (GSEA) based on GO and KEGG databases confirmed that the “osteoclast differentiation” pathway was significantly enriched among the downregulated genes (Figure 6C).

We further investigated whether LFS activates NRF2 by regulating the KEAP1‐NRF2 complex through molecular docking experiment, and conducted blind docking between LFS and KEAP1 protein through software Autodock vina. The results showed that LFS could bind to the active pocket of KEAP1 (Figure 6D), and the binding energy of multiple binding sites was predicted to be less than −4 kcal/mol, indicating a high‐affinity interaction (Figure 6E). Further, we visualized the binding target of LFS and KEAP1, and the results showed that LFS had hydrogen bonding reactions with the Gly‐367 and Val‐606 of KEAP1 (Figure 6F,G). Therefore, LFS may have the ability to bind to KEAP1 protein to activate the NRF2 pathway.

To functionally validate the essential role of NRF2 in vivo, we utilized *Nrf2* ^−/−^ mice in the experimental periodontitis model. In *Nrf2* ^−/−^ mice, the basal bone mass (*Nrf2* ^−/−^ Control) appeared lower than in WT mice. More importantly, ligation caused severe bone resorption in *Nrf2* ^−/−^ mice, and LFS treatment failed to confer any protective effect, as the bone parameters (BV/TV, Tb.Th, Tb.N, Tb.Sp) in the *Nrf2* ^−/−^ PD + LFS group were not significantly different from those in the *Nrf2* ^−/−^ PD group.

**FIGURE 7:**
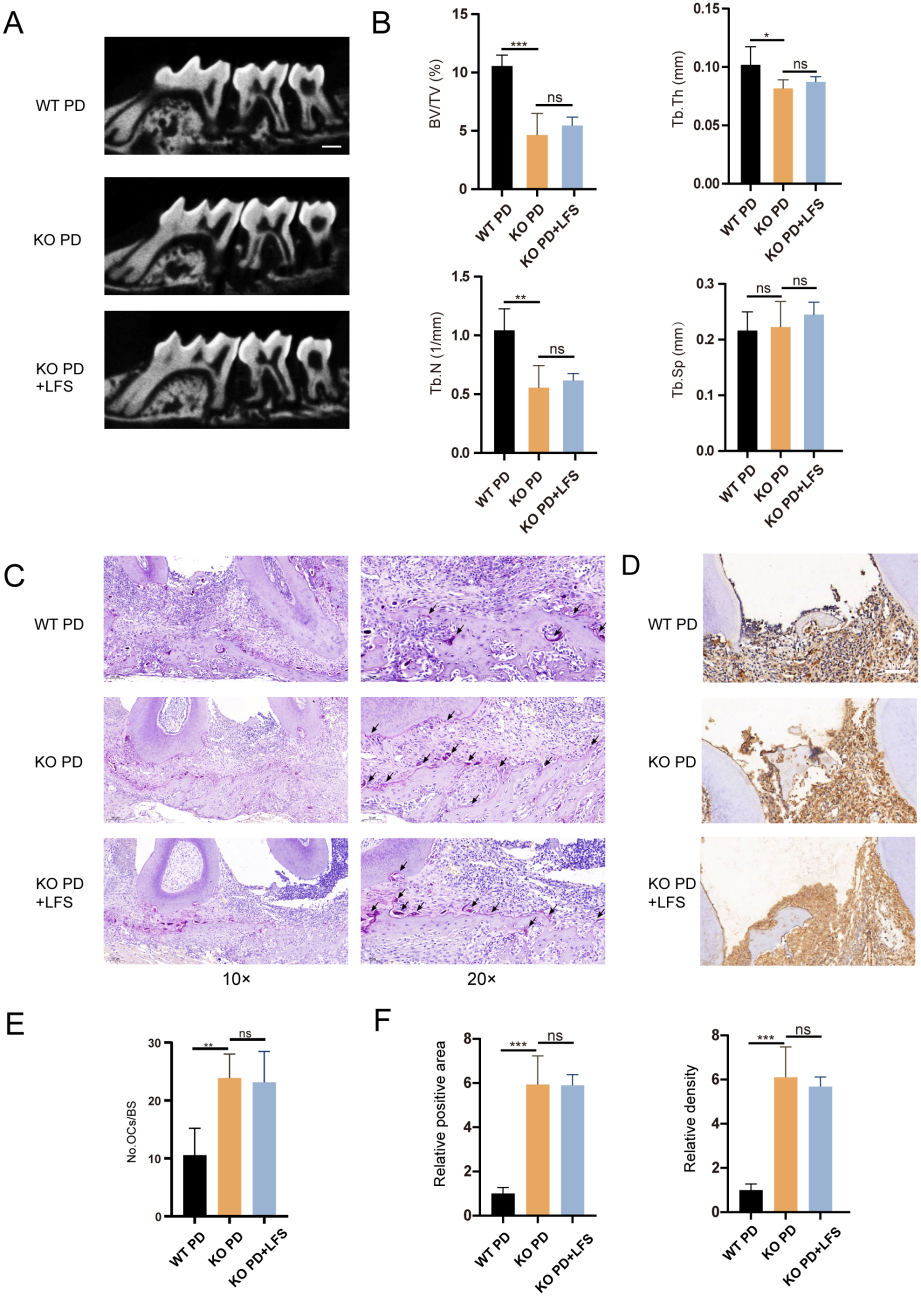
LFS mitigates alveolar bone loss and osteoclast differentiation in vivo. (A, B) Representative micro‐CT images in different groups and quantitative Analysis of bone volume per tissue volume (BV/TV) percentage, trabecular thickness (Tb.Th), trabecular number (Tb.N), and trabecular spacing (Tb.Sp) in different groups. (C, E) Representative TRAP staining images and quantitative analysis of osteoclast number in different groups in vivo. Scale bar = 100 μm. (D, F) Representative immunohistochemical images for the distribution of IL‐1β and quantitative analysis of relative mean positive area and density in different groups in vivo. Scale bar = 100 μm. *n* = 4 per group; *p < 0.05 **p < 0.01, and ***p < 0.001.

This NRF2‐dependent effect was consistent at the cellular and inflammatory levels. TRAP staining showed a high number of osteoclasts in both the *Nrf2* ^−/−^ PD and *Nrf2* ^−/−^ PD + LFS groups, with no significant difference between them (Figure 7C,E). Similarly, the elevated expression of IL‐1β in the periodontal tissues of *Nrf2* ^−/−^ PD mice was not reduced by LFS treatment (Figure 7D,F). These results unequivocally demonstrate that the ability of LFS to mitigate inflammation and osteoclastogenesis in periodontitis is strictly dependent on the presence of a functional NRF2 pathway.

### 3.6 LFS Inhibits the Growth, Virulence, and Biofilm Formation of *P. gingivalis*

**FIGURE 8:**
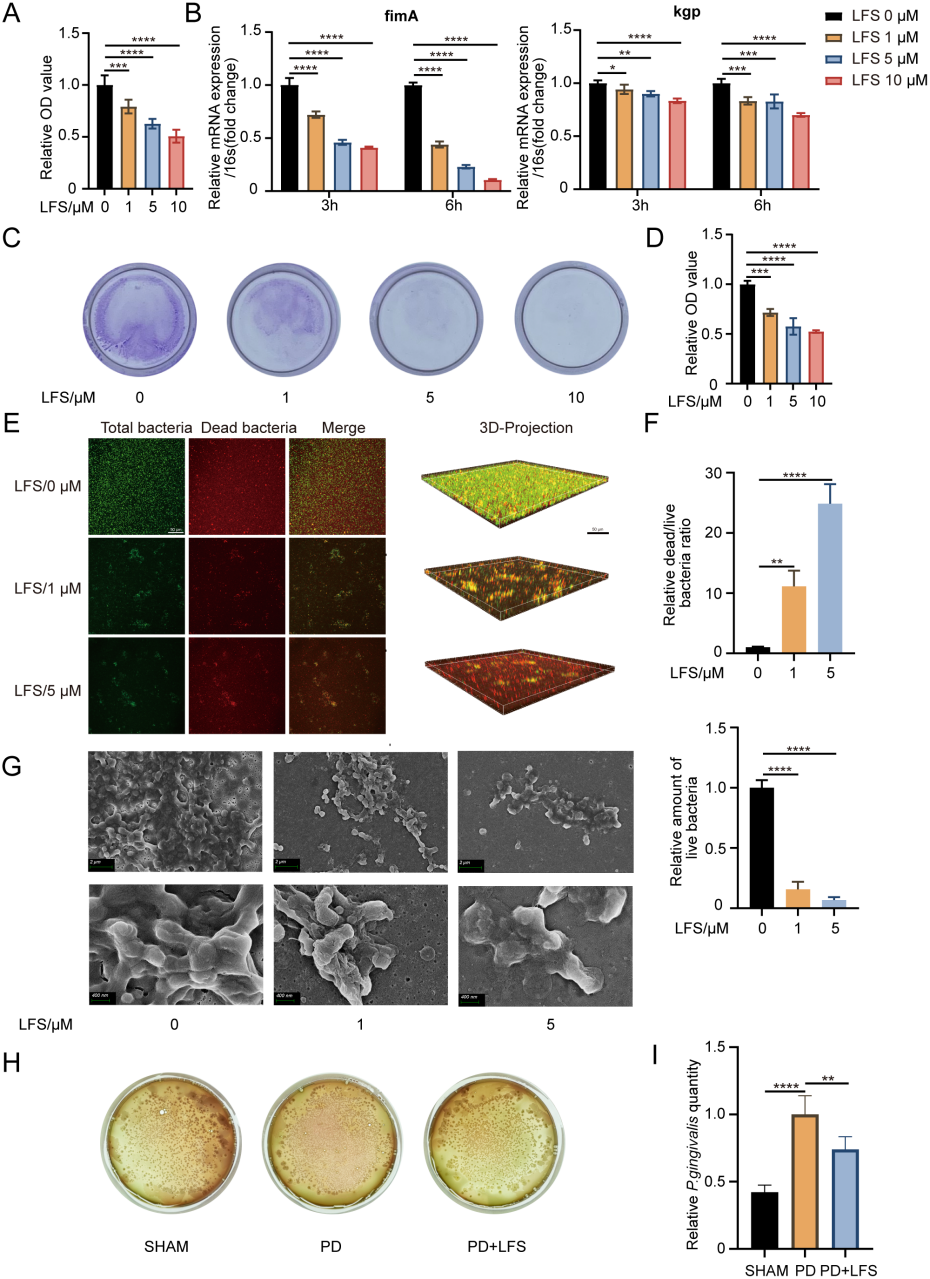
Inhibitory effect of LFS on *P. gingivalis* growth. (A) Relative absorbance of planktonic *P. gingivalis* with different concentration of LFS. (B) Relative mRNA expressions of Kgp and FimA in *P. gingivalis* with different concentration of LFS by qPCR. (C, D) Representative images and quantitative analysis of crystal violet staining of *P. gingivalis* biofilm. (E, F) Representative confocal images and quantitative analysis of biofilm formation in the presence of LFS (0, 1, and 5 μM). Scale bar = 50 μm. (G) Representative scanning electron microscope images of *P. gingivalis* biofilm formation in the presence of LFS (0, 1, and 5 μM). Scale bar = 2 μm and 400 nm respectively. (H, I) Representative images of *P. gingivalis* cultured from gingival crevicular fluid in different groups and quantitative analysis of colony count. Data are presented as means ± SD of 3 independent experiments; *p < 0.05, **p < 0.01, ***p < 0.001, and ****p < 0.0001.

Given the pivotal role of *P. gingivalis* in periodontitis pathogenesis, we investigated the direct antibacterial effects of LFS (Hussain et al. 2015). In this study, planktonic *P. gingivalis* was cultured and subjected to varying doses of LFS (0, 1, 5 and 10 μM). After 24 h, absorbance measurements demonstrated a concentration‐dependent inhibition of *P. gingivalis* growth (Figure 8A). The 1, 5 and 10 μM LFS treatments resulted in significant growth suppression compared to the untreated control (0 μM).

Furthermore, qPCR analysis revealed that LFS significantly downregulated the expression of two key virulence factors of *P. gingivalis* , FimA and Kgp (Figure 8B). This suggests that LFS can attenuate the pathogenicity of *P. gingivalis* beyond merely inhibiting its growth.

The effect of LFS on biofilm formation, a critical step in periodontal pathogenesis, was assessed by crystal violet staining (Figure 8C,D). Biofilm biomass was significantly reduced in the groups treated with 1, 5 and 10 μM LFS compared to the control group. Additionally, CLSM using a live/dead bacterial viability stain provided further visual and quantitative evidence (Figure 8E,F). The 3D architecture of the biofilm was disrupted by LFS, with a noticeable decrease in total biomass and a significant reduction in the ratio of live (green) to dead (red) bacteria, indicating a bactericidal effect. A more direct evaluation of LFS's impact on *P. gingivalis* was achieved through scanning electron microscopy, which revealed significant alterations in the number and appearance of *P. gingivalis* under LFS intervention (Figure 8G).

Finally, to confirm the antibacterial effect in vivo, we cultured bacteria from the gingival crevicular fluid of mice. The number of *P. gingivalis* colonies recovered from the PD + LFS group was significantly lower than that from the PD group (Figure 8H,I), demonstrating that LFS treatment effectively reduces the subgingival abundance of *P. gingivalis* in a living organism.

**FIGURE 9:**
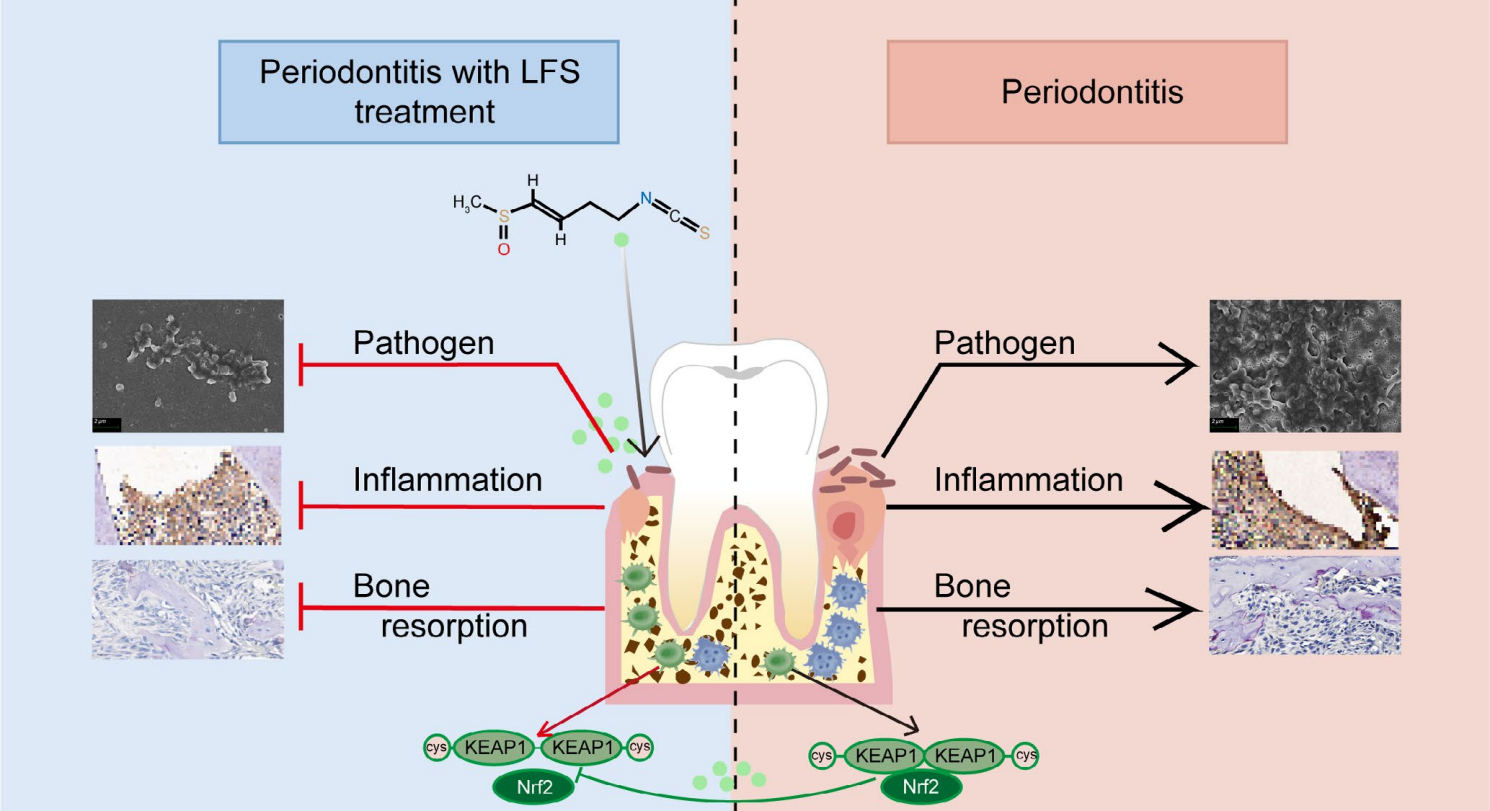
Schematic diagram of the mechanism of inhibitory effects on periodontitis by LFS.

Overall, above findings suggested that LFS activates the NRF2 pathway, and exhibited potent effects on multi‐stage of periodontitis development, making it a promising candidate for periodontitis therapy (Figure 9).

## 4 Discussion

Current clinical management of periodontitis, which primarily relies on mechanical debridement and antibiotics, often yields limited success (Costa et al. 2015; Oliveira Costa et al. 2011). These strategies predominantly target the bacterial etiology but fail to directly control the persistent host inflammatory response that drives tissue destruction and alveolar bone loss (Graziani et al. 2017; Slots 2017). This critical therapeutic gap underscores the urgent need for multi‐targeted agents capable of simultaneously mitigating the bacterial challenge, dampening the host's inflammatory response, and protecting the underlying bone architecture. Here, we identify LFS, a plant‐derived bioactive compound, as a multi‐targeted therapeutic agent. LFS directly inhibits *P. gingivalis* , suppresses the inflammatory cascade in gingival fibroblasts, and potently blocks osteoclastogenesis.

Inflammatory bone resorption often arises from the disruption of the delicate balance between osteoblasts and osteoclasts, and serves as a major driver of various bone loss disorders (Novack and Teitelbaum 2008; Rachner et al. 2011). Notably, our data demonstrate LFS neutralizes the strong pro‐osteoclastogenic effect of IL‐1β—a key inflammatory cytokine in periodontitis (Cheng et al. 2020). Moreover, unlike conventional anti‐resorptive agents such as bisphosphonates that can suppress osteoblast activity, LFS does not impair the osteogenic differentiation of human PDLCs (Imai et al. 2013). We hypothesize that this resilience may be linked to NRF2's known ability to interfere with NF‐κB signaling (Wardyn et al. 2015), a primary pathway through which IL‐1β promotes osteoclastogenesis. This positions LFS not merely as an anti‐osteoclastogenic agent, but as a potential restorer of the osteoblast–osteoclast balance under inflammatory duress.

The core mechanistic insight from our study lies in the identification of the KEAP1–NRF2 axis as the primary pathway mediating the bone‐protective and anti‐inflammatory actions of LFS. Our transcriptomic analysis provided the initial clue, revealing an LFS‐driven upregulation of *Nfe2l2* (encoding NRF2) coupled with a coordinated downregulation of osteoclast‐specific genes. Molecular docking simulations offer a structurally plausible model for this activation, predicting that LFS binds with high affinity to the KEAP1 protein, forming specific hydrogen bonds with key residues. This binding mode is characteristic of electrophilic NRF2 activators that disrupt the KEAP1‐NRF2 interaction, thereby stabilizing NRF2 and facilitating its nuclear translocation (Yamamoto et al. 2018; Itoh et al. 1999). The essential role of this pathway was confirmed in *Nrf2* ^−/−^ mice, where LFS failed to prevent bone loss or suppress osteoclastogenesis and inflammation. Future studies employing co‐immunoprecipitation or cellular thermal shift assays will be essential to directly validate the physical LFS–KEAP1 interaction and to quantify NRF2 stabilization at the protein level.

Beyond its host‐modulatory capacity, our study reveals a previously underappreciated direct antibacterial activity of LFS against *P. gingivalis* . Importantly, LFS treatment led to the downregulation of the virulence genes *fimA* and *kgp*. Given that FimA is critical for bacterial adhesion and colonization, and Kgp is a major driver of tissue degradation and immune subversion, their suppression suggests that LFS attenuates *P. gingivalis* pathogenicity beyond a mere bacteriostatic effect (Hajishengallis 2015; Olsen and Yilmaz 2016). LFS may do more than just reduce bacterial load; it may attenuate the pathogen's capacity to trigger a destructive host response. This is conceptually aligned with the “keystone pathogen” hypothesis (Hajishengallis et al. 2012), where targeting the virulence of a few critical species can have a disproportionate benefit on the entire microbial community. The reduction in subgingival *P.gingivalis* abundance in vivo confirms this ecological impact. Nevertheless, an intriguing question for future studies is whether LFS's host‐directed NRF2 activation and its direct antibacterial effects are synergistic. For instance, could a reduction in bacterial‐driven oxidative stress via NRF2 activation create an environment less favorable for the survival of anaerobic pathogens like *P. gingivalis* ?

It is important to acknowledge certain limitations of the present study. Although IL‐1β stimulation provides a controlled and biologically relevant inflammatory model, the in vivo periodontal milieu represents a far more complex network of cytokines, chemokines, and dynamic cellular interactions (Cheng et al. 2020; Garlet 2010). Future investigations employing more sophisticated in vitro systems—such as bacterial‐stimulated co‐culture models incorporating multiple host cell types—would yield a more comprehensive understanding of the immunomodulatory spectrum of LFS. Furthermore, while we have established the necessity of NRF2, the specific downstream antioxidant and cytoprotective targets that translate NRF2 activation into the observed phenotypic outcomes remain to be fully elucidated.

Despite these limitations, the protective efficacy of LFS was robustly validated in our preclinical mouse model of periodontitis. To bridge the translational gap between these promising findings and clinical application, future research should focus on developing advanced local delivery systems for LFS. Formulating LFS within bioadhesive hydrogels, polymeric nanoparticles, or biodegradable periodontal chips could enable sustained and targeted release within the periodontal pocket, thereby enhancing local bioavailability while minimizing systemic exposure (Fenton et al. 2018; Luan et al. 2023). Such advanced delivery platforms hold the key to unlocking the full therapeutic potential of LFS and translating this multi‐targeted agent into a novel clinical modality for periodontitis.

## 5 Conclusions

In summary, our findings indicate that LFS, an herbal extract, exhibits pleiotropic effects throughout various stages of periodontitis progression and ultimately preventing alveolar bone loss. It directly inhibits *P. gingivalis* , suppresses host inflammation, and attenuates osteoclastogenesis, ultimately preserving alveolar bone. This multi‐targeted efficacy, hinged on NRF2 pathway activation, presents LFS as a compelling candidate for a novel, integrated therapeutic strategy against periodontitis.

## Author Contributions

**Hantao Yao:** formal analysis (equal), investigation (equal), methodology (equal), validation (equal), writing – original draft (equal). **Kai Yu:** formal analysis (equal), investigation (equal), methodology (equal), validation (equal), writing – original draft (equal). **Bulin Jiang:** data curation (supporting), investigation (supporting). **Yilin Liao:** data curation (supporting), investigation (supporting). **Yaoyu Zhao:** data curation (supporting), investigation (supporting). **Jingqiu Chen:** data curation (supporting), investigation (supporting). **Ting Li:** data curation (supporting), investigation (supporting). **Mengjie Yin:** data curation (supporting), investigation (supporting). **Yue Sheng:** data curation (supporting), investigation (supporting). **Wengwanyue Ye:** data curation (supporting), investigation (supporting). **Boxuan Zhao:** data curation (supporting), investigation (supporting). **Minquan Du:** conceptualization (equal), funding acquisition (equal), resources (equal), supervision (equal), writing – review and editing (equal). **Yaoting Ji:** conceptualization (equal), funding acquisition (equal), resources (equal), supervision (equal), writing – review and editing (equal).

## Funding

This work was supported by the National Natural Science Foundation of China (grant numbers 82372463, 82172493, and 81771084), Natural Science Foundation of Zhejiang Province (QN26H140018), China Postdoctoral Science Foundation (2024M762879), Zhejiang Province Traditional Chinese Medicine Science and Technology Project (2026ZL0481).

## Ethics Statement

The collection of clinical specimens was approved by the Medical Ethics Committee of Wuhan University School of Stomatology (authorized B16/2021). Informed consent was given to all patients.

## Conflicts of Interest

The authors declare no conflicts of interest.

## Data Availability

The data utilized in this study can be obtained from the corresponding author upon reasonable inquiry.
